# The Interaction Between the Nematode *Caenorhabditis elegans* and Its Coexisting Fungal Microbiome Member *Barnettozyma californica*

**DOI:** 10.1111/1758-2229.70177

**Published:** 2025-08-19

**Authors:** Carola Petersen, Hanne Griem‐Krey, Christina Martínez Christophersen, Hinrich Schulenburg, Michael Habig

**Affiliations:** ^1^ Evolutionary Ecology and Genetics Christian‐Albrechts University of Kiel Kiel Germany; ^2^ Fungal Evolutionary Genetics Christian‐Albrechts University of Kiel Kiel Germany; ^3^ Max‐Planck Institute for Evolutionary Biology Ploen Germany

**Keywords:** bacteria, Caenorhabditis elegans, fungi

## Abstract

The nematode *Caenorhabditis elegans* is known to feed on and interact with bacteria in its environment and has become a model organism for microbiome studies. However, whether and how *C. elegans* interacts with co‐occurring fungi remains largely unknown, despite the presence of many fungal species in its natural habitat. Here, we isolate the yeast *Barnettozyma californica* from a mesocosm experiment with *C. elegans* and characterise its genome and interaction with the nematode. We find that, like bacterial microbiota, *B. californica* can colonise the intestine of *C. elegans* and can serve as a sole, albeit poor, food source for adult nematodes. Yet, when present together with *Escherichia coli* OP50, the fungus can lead to higher population growth and altered foraging behaviour, suggesting a context‐dependent benefit. This effect varied between different natural *C. elegans* strains, suggesting a genomic basis for the nematode's interaction with *B. californica* . On the fungal side, we could not identify any obvious candidate genes for its interaction with *C. elegans* and/or *E. coli* OP50, despite obtaining a fully assembled and annotated genome of the isolated *B. californica* strain. Overall, our results provide an intriguing example of the complexity and multi‐level relationship between naturally interacting fungi, bacteria and animals.

## Graphical abstract

*Barnettozyma californica* is a member of the fungal microbiome of the model nematode *Caenorhabditis elegans* and can serve as food for worms. When combined with *E. coli* , this fungus can increase population growth and reduce foraging behaviour dependent on *C. elegans* genotype, revealing a genetic influence on host‐fungus‐bacterium interactions.

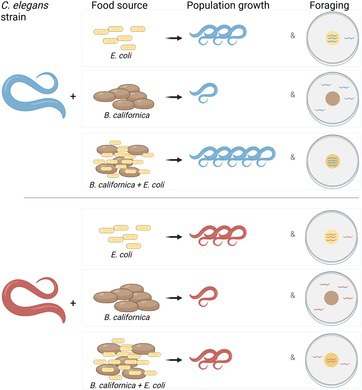

## 1 Introduction

*Caenorhabditis elegans* inhabits a diverse microbial community in its natural environment, including bacteria and fungi. These microbes play various roles in the life cycle of *C. elegans* —serving as food, competitors, commensals or pathogens—and can also be part of its microbiome. The interaction of *C. elegans* with pathogens such as the bacteria *Bacillus thuringiensis* , *Pseudomonas aeruginosa* , microsporidia of the genus *Nematocida*, the oomycete *Myzocytiopsis humicola* or the Orsay virus has been studied over the last decades (Tan et al. 1999; Troemel et al. 2008; Schulte et al. 2010; Félix et al. 2011; Osman et al. 2018; Tecle and Troemel 2022; Tran and Luallen 2024; Zárate‐Potes et al. 2024). More recently, interest has expanded to include the worm's bacterial microbiome, with the first studies published in 2016 (Berg et al. 2016; Dirksen et al. 2016; Samuel et al. 2016; Schulenburg and Félix 2017). These and subsequent studies have shown that the bacterial microbiome of *C. elegans* is distinct from its surrounding microbial environment under both natural and experimental conditions (Berg et al. 2016; Dirksen et al. 2016, 2020; Samuel et al. 2016; Petersen et al. 2023; Zimmermann et al. 2024; Johnke et al. 2025). In *C. elegans* , unclassified Enterobacteriaceae are the most common bacteria, along with members of the genera *Pseudomonas*, *Stenotrophomonas*, *Ochrobactrum* and *Sphingomonas* (Zhang et al. 2017). The composition, assembly, stability and variation of the microbiome are influenced by the host's developmental stage and genotype (Dirksen et al. 2016; Zhang, Weckhorst, et al. 2021; Zimmermann et al. 2024). The majority of bacterial strains isolated from either the worm's habitat or directly from its intestine support robust *C. elegans* growth. However, some can be pathogenic (Samuel et al. 2016; Griem‐Krey et al. 2023; Gonzalez and Irazoqui 2024). To better understand the influence of microbial communities on the host, a simplified synthetic 12‐member microbiota community, known as the CeMbio resource, was developed (Dirksen et al. 2020). All CeMbio bacteria can colonise the *C. elegans* intestine, either as a community or individually, to varying degrees. Most strains accelerate the worm's development compared to those grown on standard lab food, *Escherichia coli* OP50 (Dirksen et al. 2020). However, some CeMbio bacteria shorten the lifespan of immunodeficient animals (Gonzalez and Irazoqui 2024). Beneficial effects, such as the protective role of *Pseudomonas lurida* MYb11 against the pathogen *B. thuringiensis* Bt679, can be disrupted by p38 MAPK signalling inhibition. This suggests that microbiota effects on *C. elegans* can be context‐dependent (Kissoyan et al. 2019, 2022; Griem‐Krey et al. 2023; Pees et al. 2024).

Like bacteria, many fungi are found in the natural habitat of *C. elegans* (Dirksen et al. 2016). In contrast to its interactions with naturally co‐occurring bacteria, very little is known about its interactions with co‐occurring fungi. The focus has primarily been on pathogenic interactions. Spores of *Drechmeria coniospora* and other natural fungal endoparasites of *C. elegans* , such as *Hirsutella rhossiliensis*, *Haptoglossa dickii* and *Catenaria anguillulae* , infect nematodes and complete their vegetative life cycle within infected hosts. Upon infection, *D. coniospora* produces enterotoxins in the epidermis of *C. elegans* , which manipulate the host's immune responses (Zhang, Harding, et al. 2021). Additionally, *C. elegans* has been used as a model organism to study host immune responses towards fungal human pathogens (Madende et al. 2020). Innate immune responses of *C. elegans* towards fungal pathogens involve antimicrobial peptides, lectins, lysozymes, the p38 and ERK mitogen‐activated protein kinase pathways, as well as the DAF‐2 and TGF‐ß/DBL‐1 signalling pathways (Zugasti and Ewbank 2009; Zugasti et al. 2016). Moreover, *C. elegans* produces chemical compounds with antifungal activity, making it a valuable infection model (Breger et al. 2007; Madende et al. 2020). A completely different type of interaction between fungi and nematodes involves nematode‐trapping fungi, carnivorous microorganisms that capture and digest nematodes using specialised trapping structures (Jiang et al. 2017). These include *Arthrobotrys* spp. and *Drechslerella stenobrocha*, which ensnare nematodes with constricting ring traps or adhesive traps (Yang et al. 2007; Nordbring‐Hertz et al. 2011; Liu et al. 2012), highlighting the diverse range of possible interactions between fungi and nematodes.

Apart from these, few well‐characterised interactions between fungi and nematodes, and despite the fact that fungi have been isolated directly from natural *C. elegans* strains (Dirksen et al. 2016), little is known about fungi as members of the nematode's microbiome. In a recent study examining *C. elegans* in laboratory compost mesocosms, several fungal species were identified, with *Barnettozyma californica* strains being particularly abundant in the worms (Petersen et al. 2023). Interestingly, worm populations that lived for multiple generations in a microbial community that included *Barnettozyma* showed a particularly high fitness. This raises the question of whether the observed fitness advantage was directly caused by *Barnettozyma* fungi and whether this fungal taxon plays an important role for *C. elegans* —either as a food source or as a member of its microbiota.

*Barnettozyma* is a genus in the order Phaffomycetales, which was established in 2023 based on a phylogenetic analysis of 290 BUSCO genes (Groenewald et al. 2023). The genus *Barnettozyma* was originally described in 2008 based on a multigene phylogenetic analysis, which included *B. californica* (previously known as *Williopsis californica*) (Kurtzman et al. 2008). *B. californica* has a worldwide distribution (Kurtzman 2011), with its type strain CBS 252 first isolated from soil in California in 1931 (Lodder 1932). Although relatively few genomic sequences are available for *B. californica* , the first draft genomes—produced using short‐read sequencing technologies—were obtained from two *B. californica* isolates from Ireland (Mullen et al. 2018). To date, no chromosome‐level genome assemblies exist for this species. *B. californica* has demonstrated the ability to grow on xylose and can utilise a broader range of carbon sources than other tested members of the *Barnettozyma* genus. It also exhibits ammonium reduction capabilities and can utilise both nitrite and nitrate (Falih and Wainwright 1995; Kurtzman 2011; Shen et al. 2018). Additionally, *B. californica* strain K1, isolated from surface sediments of the Pearl River, has been shown to perform heterotrophic nitrification‐aerobic denitrification (Fang et al. 2021). Recently, another member of the *Barnettozyma* genus, *B. botsteinii*, was discovered in the intestinal tract of the termite *Macrotermes bellicosus*. Like *B. californica* , *B. botsteinii* can grow on xylose, and its ability to degrade plant‐derived polymers suggests a potential role in assisting termite metabolism (Arrey et al. 2021). In summary, despite its wide distribution and presence in various environments, many aspects of *B. californica* 's life history and genomic characteristics remain to be explored. Additionally, how *B. californica* interacts with *C. elegans* and whether and how it directly affects the nematode's fitness is unknown.

Here, we characterise the interaction between *B. californica* and *C. elegans* and show that *B. californica* can colonise the nematode's intestine and influence its behaviour. When adult worms are fed *B. californica* as their sole food source, it proves less beneficial than bacterial food and cannot support the full development of early larvae. In contrast, when *B. californica* is offered as food in combination with bacteria—presumably more representative of *C. elegans* ' natural environment—it can increase *C. elegans* population growth; though this effect depends on the nematode's genotype. Thus, we demonstrate that a fungus co‐occurring with *C. elegans* can influence the worm's fitness.

## 2 Results

We first isolated *B. californica* MYf642 from a mesocosm experiment and characterised its interaction with *C. elegans* . We then analysed the genome of *B. californica* MYf642 to determine whether it reflects its interaction with *C. elegans* .

### 2.1 *B. californica* MYf642 Can Colonise *C. elegans* Adults

**FIGURE 1:**
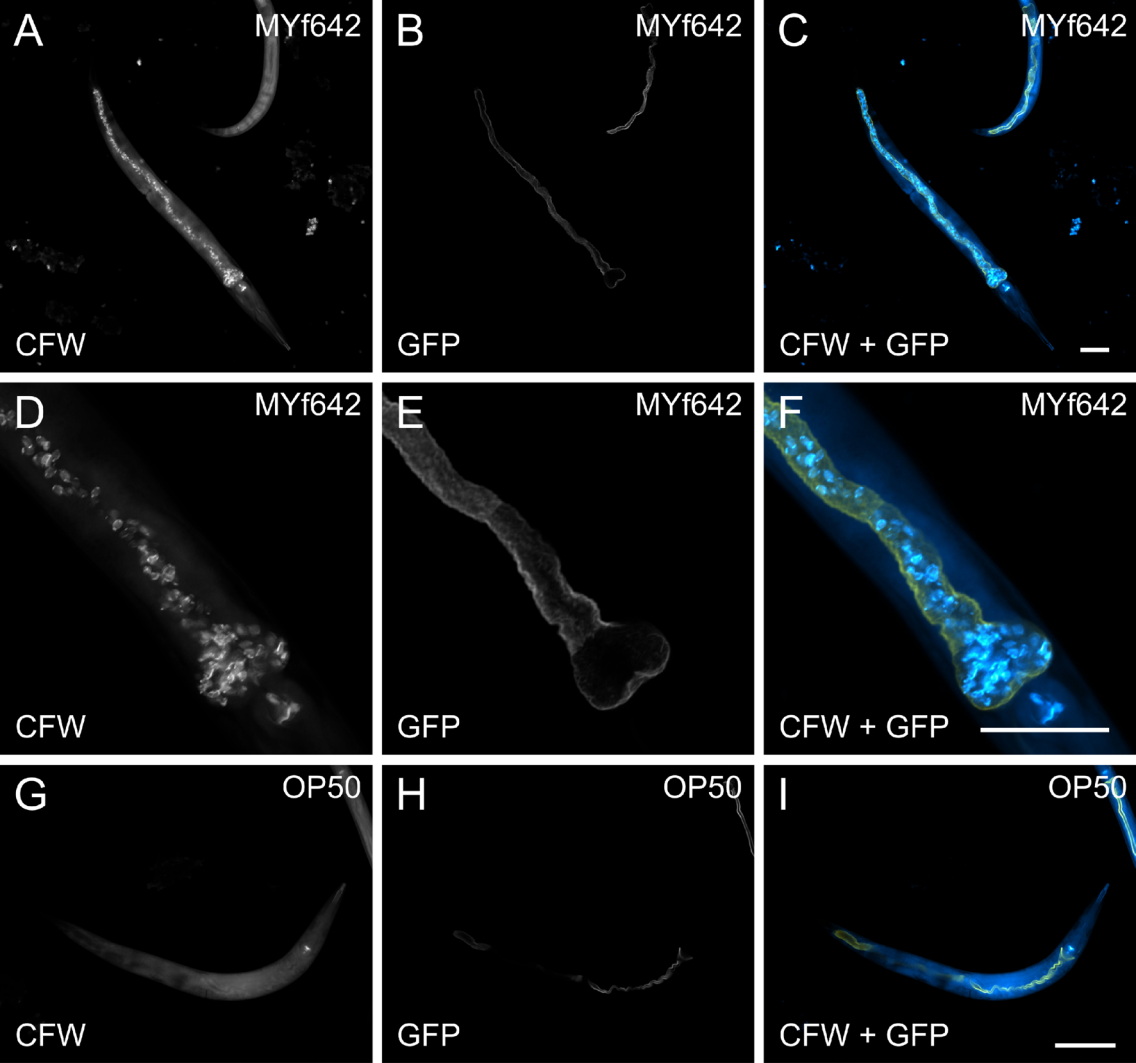
*C. elegans* ingest *Barnettozyma californica* MYf642. (A–F) *C. elegans* dkIs37[*act‐5p::GFP::pgp‐1*] can ingest *B. californica* MYf642, with fungal cells observed throughout the intestine (A, C, D, F) (white or light blue). (G–I) *E. coli* OP50 cells do not contain chitin and hardly pass the worm grinder and are therefore not stained by the calcofluor white staining. The apical intestinal membrane of *C. elegans* dkIs37[*act‐5p::GFP::pgp‐1*] expresses GFP and is shown in white (B, E, H) or yellow (C, F, I). Calcofluor white (CFW) stained *B. californica* MYf642 is depicted in white (A, D) or light blue (C, F). Worms were visualised using confocal laser scanning microscopy. Scale bars represent 50 μm. Images were false‐coloured using ImageJ.

Worms exposed to the microbiome of a specific compost mesocosm consistently showed a higher relative abundance of ASVs from the fungal genus *Barnettozyma* (Petersen et al. 2023). We isolated *B. californica* MYf642 from this mesocosm and examined whether it is capable of colonising the *C. elegans* intestine. To visualise *B. californica* MYf642 in *C. elegans* , we used calcofluor white staining, which specifically binds to chitin. We also used the *C. elegans* strain dkIs37[*act‐5p::GFP::pgp‐1*], in which the intestinal basal membrane is fluorescently labelled to aid localisation. Our results show that *B. californica* MYf642 colonises *C. elegans* adults, with fungal cells observed throughout the whole intestine, posterior to the grinder (Figure 1A–F). Additionally, we observed and documented the feeding behaviour of *C. elegans* , including ingestion, passage through the worm intestine and excretion of *B. californica* MYf642 (Movies S1–S2).

**FIGURE 2:**
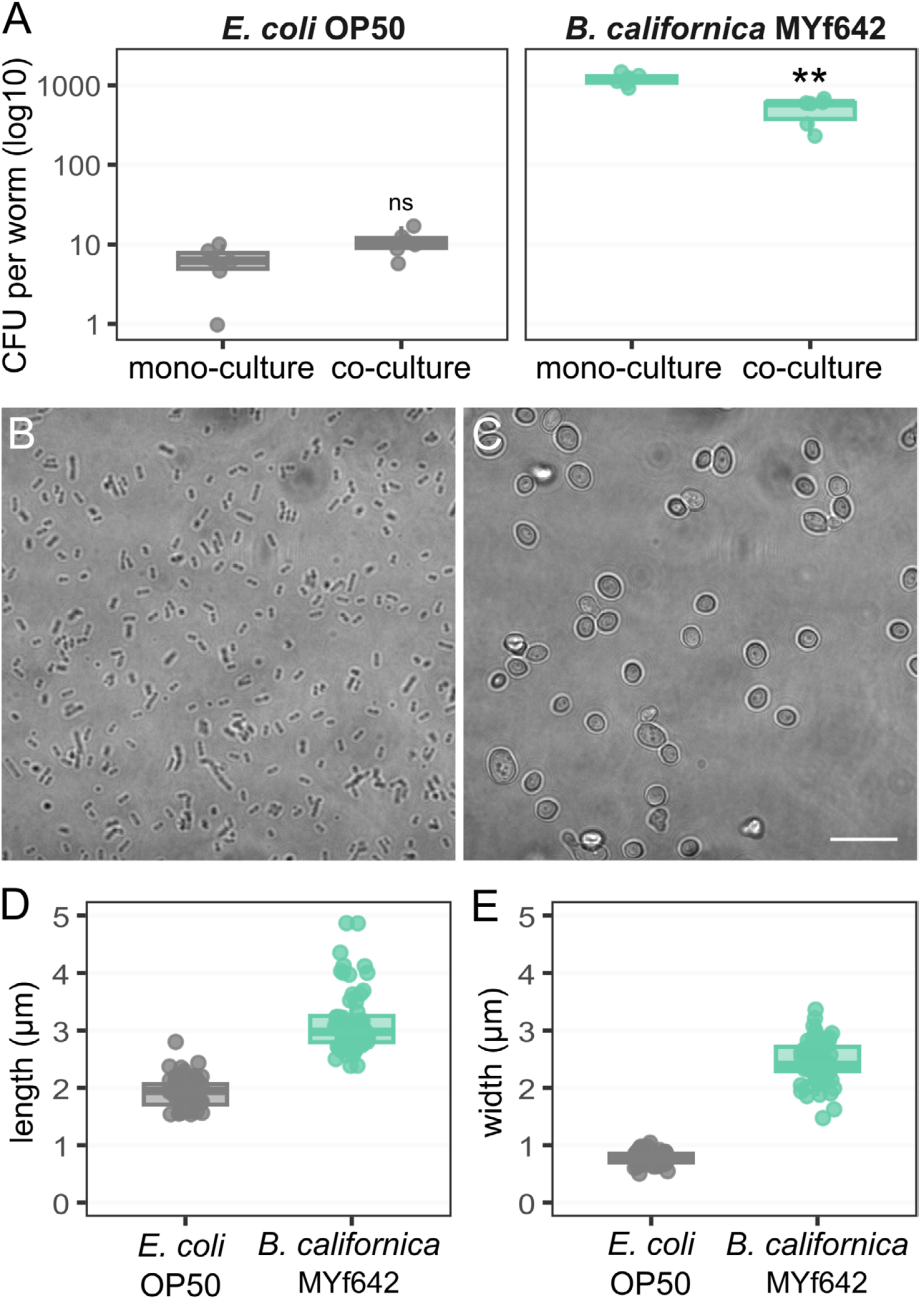
*B. californica* MYf642 colonises the intestine of *C. elegans* and displays larger cell size compared to *E. coli* OP50. (A) Intestinal colonisation of *C. elegans* N2 as colony forming units (CFU) per worm fed for 24 h with either *E. coli* OP50 (grey), *B. californica* MYf642 (light green) or a combination of both microbes (co‐culture). The y‐axis is log10‐transformed to visualise differences in colonisation levels. Asterisks indicate significant differences between mono‐ and co‐cultures (determined by Wilcoxon rank sum test with FDR‐correction for multiple testing), *p* < 0.01 (**), ns = non‐significant, *n* = 6. Micrographs of (B) *E. coli* OP50 and (C) *B. californica* MYf642, scale bar = 10 μm. (D) Cell length and (E) cell width of *E. coli* OP50 (grey) in comparison to *B. californica* MYf642 (light green).

Control worms fed solely on *E. coli* OP50 did not show any stained cells in their intestine because *E. coli* does not contain chitin (Figure 1G–I). Previous studies have shown that intact *E. coli* cells are rarely found posterior to the grinder in *C. elegans* (Portal‐Celhay et al. 2012). The *B. californica* cells in the worm intestine were intact as indicated by colony forming units (CFU) obtained directly from worms (Figure 2A, Table S1A). We found that *B. californica* survived the grinder significantly more than OP50 (Wilcoxon rank sum test with *p* = 0.004 in mono‐culture, Wilcoxon signed rank test with *p* = 0.042 in co‐culture). *B. californica* and OP50 fed as a mixture reduced *B. californica* uptake (Wilcoxon rank sum test with *p* = 0.004), whereas uptake of OP50 was not affected (Table S2A). The high number of alive *B. californica* MYf642 cells inside the gut of *C. elegans* may account for its wider appearance compared to the gut in the OP50 fed control. Next, we compared the cell size of *B. californica* MYf642 to that of *E. coli* OP50. The average cell dimensions of *B. californica* MYf642 (mean length = 3.155 μm ± 0.561 SD; width = 2.457 μm ± 0.375 SD) were substantially larger than those of *E. coli* OP50 (length = 1.922 μm ± 0.258 SD; width = 0.783 μm ± 0.110 SD) (Figure 2B–E, Table S1B).

### 2.2 *B. californica* MYf642 Is a Poor Food Source for *C. elegans* Alone, but Can Significantly Increase the Fitness When Presented Alongside Bacterial Food

**FIGURE 3:**
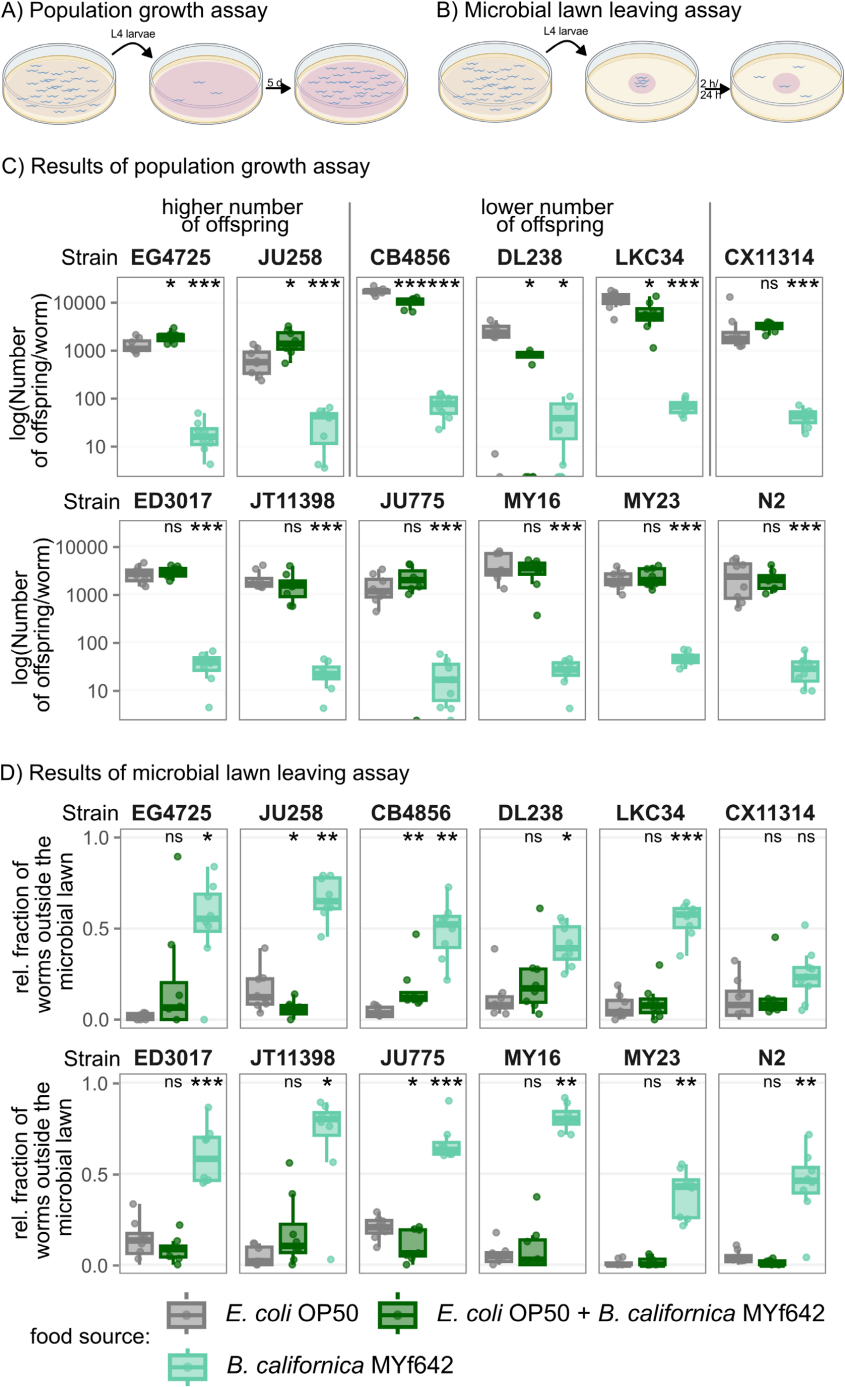
*B. californica* MYf642 in combination with *E. coli* OP50 as food results in a worm strain‐dependent effect on the number of *C. elegans* offspring and behavioural response. (A) Outline of the population growth assay: Three synchronised L4 larvae are transferred onto a microbial lawn and the population size is evaluated after 5 days. (B) Outline of the microbial lawn leaving assay: Approximately 30 synchronised L4 larvae are put onto a central microbial lawn and after 2 h and 24 h the fraction of nematodes outside the microbial lawn is determined. (C–D) The figure shows the effect on population growth (C) and the behavioural response (D) on *E. coli* OP50 (grey), *E. coli* OP50 combined with *B. californica* MYf642 (dark green) and *B. californica* MYf642 alone (light green). *B. californica* MYf642 as the sole food source results in significantly lower number of offspring per worm and a higher number of worms outside the microbial lawn after 24 h across all worm strains (compared to *E. coli* OP50 as sole food source). When combined with *E. coli* OP50, *B. californica* MYf642 leads to a higher number of offspring in *C. elegans* strains EG4725 and JU258, while significantly lowering the number of offspring in strains CB4856, DL238 and LKC34. In JU258 and CB4856, this is associated with fewer or more worms leaving the microbial lawn, respectively. The y‐axis is log10‐transformed to visualise differences in population growth levels. Significant differences (determined by the Wilcoxon rank sum test with Holm correction for multiple testing) compared to *E. coli* OP50 as the sole food source are indicated as follows: *p* < 0.05 (*), *p* < 0.01 (**), *p* < 0.001 (***), ns = non‐significant. *n* = 8 biological replicates.

Next, we tested whether *B. californica* can serve as a sole food source or, in combination with the standard laboratory food source *E. coli* OP50, influence the fitness of *C. elegans* . To account for the genetic variability of *C. elegans* and avoid restricting our analysis to the canonical laboratory strain N2, we included 11 additional strains from the *C. elegans* mapping population provided by the *Caenorhabditis* Natural Diversity Resource (CaeNDR) (Cook et al. 2017; Crombie et al. 2024). In a population growth assay, three L4 larvae of each *C. elegans* strain were added to microbial lawns consisting of either *B. californica* MYf642, *E. coli* OP50 or a combination of both (which would, by containing both a fungal and a bacterial component, presumably be more akin to the microbial composition in the natural habitat of *C. elegans* ). The number of offspring was determined after five days (Figure 3A, Table S1C). For all *C. elegans* strains tested, *B. californica* MYf642 as the sole food source resulted in significantly lower population growth compared to the *E. coli* OP50 control (Figure 3C, Table S2B). On average, the number of offspring on *B. californica* ranged from 0.4% to 5.1% of the respective offspring count on *E. coli* OP50, with a maximum of an average of 79 offspring per original worm in strain CB4856 and a minimum of 20 on average in strain JU775. The low number of offspring per worm compared to on *E. coli* suggests that *B. californica* MYf642 supports only minimal development of *C. elegans* , rather than its complete life cycle, indicating it is a poor food source on its own. To evaluate whether *C. elegans* can complete its full life cycle, we tracked larval development from the L1 stage onward on *B. californica* MYf642, *E. coli* OP50 or a combination of both microbial strains. *C. elegans* N2 larvae reached adulthood within 72 h on *E. coli* OP50 alone or in combination with *B. californica* MYf642 (Figure S1A, Table S1D). In contrast, no adult worms were observed on *B. californica* MYf642 alone, even after 14 days post L1 stage.

The poor population growth of *C. elegans* on *B. californica* MYf642 alone contrasts with the results observed when the fungus was combined with bacterial food (Figure 3C). On the combined microbial lawn, for eight *C. elegans* strains, population growth was not significantly different from that on the *E. coli* OP50 control. For three other strains (CB4856, DL238 and LKC34), the number of offspring was significantly lower on the mixed microbial lawn, reaching 59%, 19% and 53% of the respective *E. coli* OP50 control levels. Most interestingly, for *C. elegans* strains EG4725 and JU258, the number of offspring was significantly higher on the mixture of *E. coli* OP50 and *B. californica* MYf642. Here, the mean number of offspring per worm was 150% and 252% of the respective *E. coli* OP50 control, indicating a strong positive effect on population growth. Using population growth as a proxy for fitness, we conclude that *B. californica* MYf642 can influence the fitness of *C. elegans* when present alongside a bacterial food source. This effect varies from positive to negative, depending on the *C. elegans* genotype.

### 2.3 *B. californica* MYf642 Affects the Behaviour of *C. elegans* in a Genotype‐Dependent Manner

Next, we hypothesised that *B. californica* might influence the exploratory behaviour of the nematode, similar to how different bacterial species affect dietary choice behaviour in *C. elegans* (Shtonda and Avery 2006; Meisel and Kim 2014). To test this, we placed approximately 30 synchronised L4 worms onto a microbial lawn consisting of either *B. californica* MYf642, *E. coli* OP50 or a combination of these two (Figure *3*B). The microbial lawn‐leaving behaviour of twelve *C. elegans* strains was evaluated by measuring the fraction of worms found outside the microbial lawn after 2 and 24 h (Figure 3D for 24 h data and Figure S1B for 2 h data, Table S1E). When placed on a *B. californica* MYf642 lawn, a significantly larger fraction of worms was found outside the microbial lawn after 24 h compared to the *E. coli* OP50 control (Table S2C). This suggests that the nematodes increase their foraging behaviour due to *B. californica* being a poor food source. This contrasts with the situation when the microbial lawn contained both the fungi and the bacteria. Here, we observed *C. elegans* genotype‐dependent variation in the response. After 24 h, a significantly higher fraction of worms left the mixed microbial lawn in strain CB4856—one of the strains that also exhibited lower population growth on these mixed microbial lawns. Interestingly, a lower fraction of worms left the mixed microbial lawn for the *C. elegans* strains JU258 (which displayed higher population growth on these mixed microbial lawns) and JU775. This suggests that, for these *C. elegans* strains, the mixed microbial lawn may provide a more favourable environment. Although there appears to be a correlation between the results of the microbial lawn‐leaving assay at 24 h and the population growth assay (Figure S2), this correlation is not statistically significant. In addition to the 24‐h time point, we also tested lawn‐leaving behaviour after 2 h. Interestingly, at this earlier time point, four *C. elegans* strains—including CB4856, which at 24 h showed a much larger fraction outside the mixed microbial lawn—remained more on the mixed microbial lawn than on the bacterial lawn. To investigate whether the previously observed differences in offspring production and lawn‐leaving behaviour of *C. elegans* JU258 are associated with microbial preference, we monitored the choice behaviour of *C. elegans* JU258 and N2 over time when given the option to choose between *E. coli* OP50 and a combination of OP50 and *B. californica* MYf642. Both worm strains showed no preference and chose both microbial lawns equally after 2, 6 and 24 h (Figure S3, Table S1F). Direct comparison of JU258 and N2 showed a difference in choice behaviour after 24 h (*p* = 0.018, Table S2D). In summary, *B. californica* MYf642 affects the foraging behaviour of *C. elegans* when mixed with *E. coli* OP50 in a genotype‐dependent manner.

### 2.4 The Genome of *B. californica* MYf642 Comprises Seven Chromosomes and One Mitochondrial Genome

**FIGURE 4:**
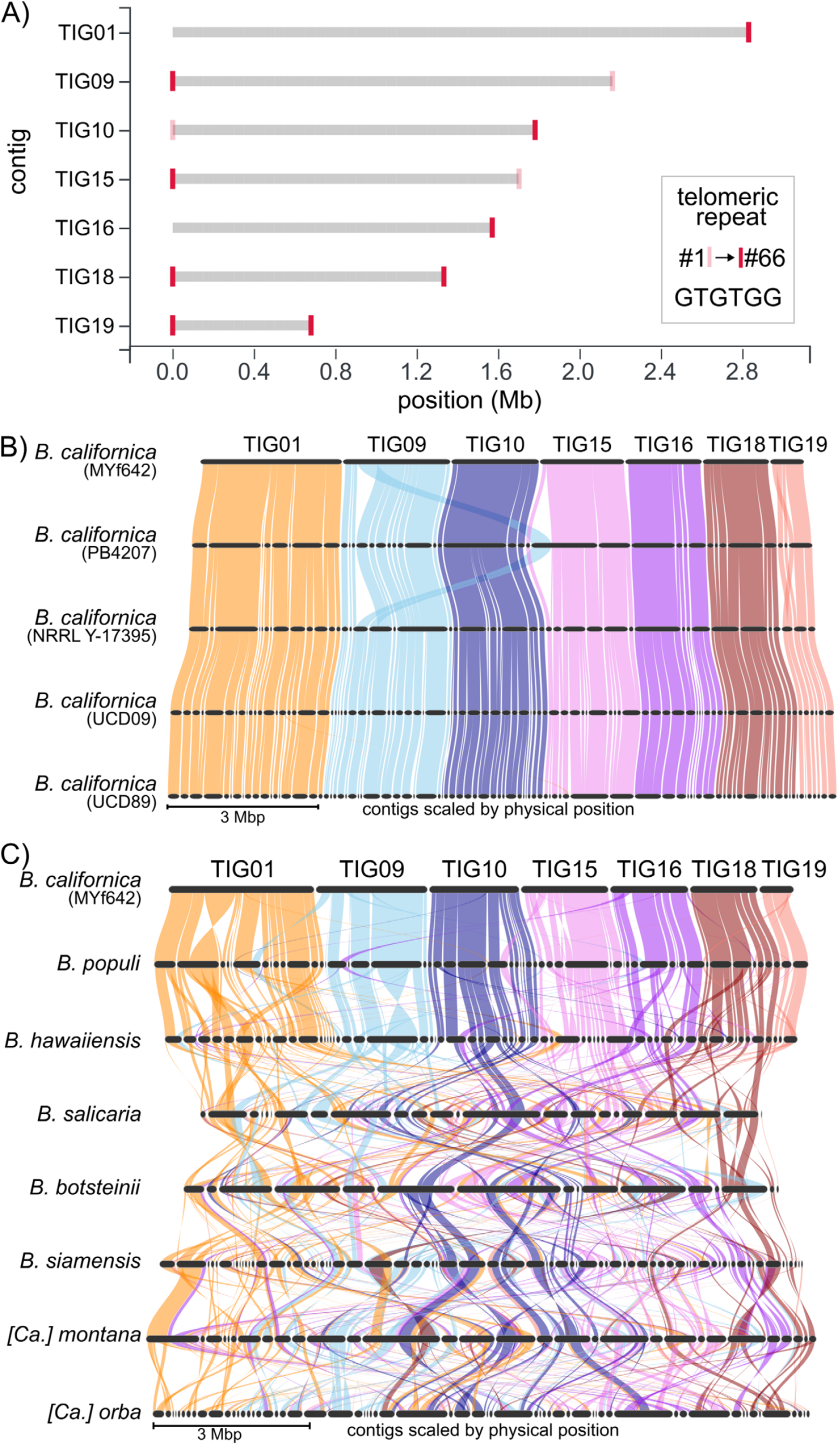
Chromosome‐level assembly of *B. californica* MYf642. (A) Tapestry report on contig sizes and the presence of telomere repeats in the seven contigs of the *B. californica* MYf642 assembly. Five of the seven contigs show telomeres at both ends. (B) Synteny plot based on orthologous genes between *B. californica* MYf642 and four publicly available fragmented assemblies of *B. californica* , showing high levels of synteny across all *B. californica* MYf642 contigs. The absence of syntenic regions spanning more than one contig or scaffold suggests that the *B. californica* MYf642 contigs represent individual chromosomes (see also Figure S4). (C) Synteny plot between *B. californica* MYf642 and other species within the *Barnettozyma* genus reveals high structural variation, which increases with phylogenetic distance.

Having established that *B. californica* MYf642 affects the fitness and behaviour of *C. elegans* , we next sequenced and assembled its genome as a resource for future experimental analyses and also to identify patterns that might help us understand its interaction with *C. elegans* . Using PacBio HiFi CLR reads, we compared three different assembly methods (see Section 4: *Methods and Materials*). The best assembly contained seven nuclear contigs and one mitochondrial genome, resulting in a total genome size of 12.1 Mb (Figure 4A, Table S3). A full genomic phylogeny based on orthologous proteins identifies the isolate MYf642 to fall within the species *B. californica* (Figure S4A). We compared the seven nuclear contigs with four publicly available, more fragmented assemblies of *B. californica* and found a high degree of synteny between orthologous genes (Figure 4B, Figure S4B,C). With one exception, no contig from the fragmented assemblies spans more than one contig of the *B. californica* MYf642 assembly. The exception is the fragmented assembly of *B. californica* strain PB4207, where one contig shows synteny with both TIG09 and TIG15 of the assembly. However, this synteny discrepancy is also present compared to the other three fragmented assemblies of *B. californica* , suggesting that it is either due to a structural variation or an assembly error in PB4207. This conclusion is supported by the presence of telomeric repeats at both ends of five of the seven contigs assembled here by us (Figure 4A). Although we did not detect the canonical 5′‐TTAGGG‐3′ telomeric repeat, we identified a novel telomeric repeat sequence, 5′‐GTGTGG‐3′, which differs from the wide variety of telomeric repeats previously described in ascomycetous yeasts, especially species within the subphylum *Saccharomycotina* (Červenák et al. 2021). The telomeric repeat sequence we identified is similar to but shorter than those described for other members of the *Phaffomycetaceae* (Červenák et al. 2021). The presence of telomeric repeats at both ends of five contigs, along with the strong synteny across all published *B. californica* genomes, supports our conclusion that *B. californica* MYf642 has seven nuclear chromosomes.

### 2.5 The Genome of *B. californica* MYf642 Is Densely Populated With Genes and Contains a Low Proportion of Repetitive and Transposable Elements

We annotated TEs, genes, tRNAs and functionally characterised putative secreted proteins, effectors, proteases and Carbohydrate‐Active Enzymes (CAZymes), along with gene‐wise relative synonymous codon usage (Tables S3 and S4). The genome of *B. californica* MYf642 contains 5893 genes, comparable to the 5802–5803 genes reported in the fragmented assemblies of *B. californica* strains UCD09 and UCD5803 (Mullen et al. 2018). The genome is gene‐dense, with genes covering 70.5% of the genome, while TEs account for a relatively low 2.1%. Of the gene transcripts, 312 contain a secretion signal, and among these, a relatively small number (78) are putative effectors—small secreted proteins that are thought to interact with potential hosts (Jones and Dangl 2006) or other microorganisms (Snelders et al. 2020). Additionally, we identified 191 putative proteases, 143 putative CAZymes and a total of 428 TEs and repeats (Table S4). Among the TEs, *hAT* transposons, a widespread class II DNA transposon (Atkinson 2015), are the most frequent in the *B. californica* MYf642 genome (Table S5). Furthermore, we functionally annotated all transcripts using Blast2GO (Table S6).

### 2.6 The Genome of *Barnettozyma californica* MYf642 Is Similar to Other Members of the *Barnettozyma* Genus

**FIGURE 5:**
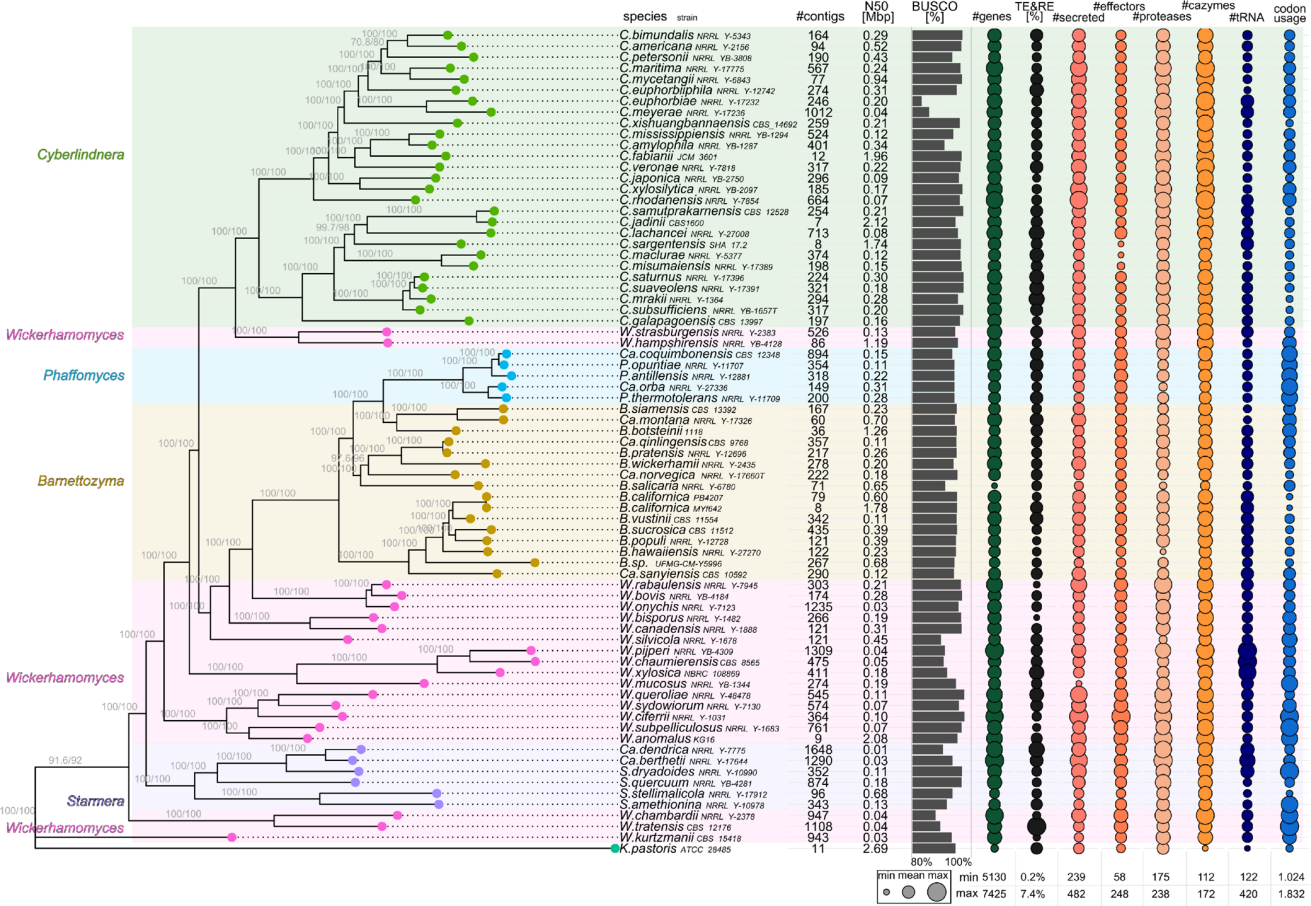
Phylogeny and summary of functional annotation of Phaffomycetales. Left: Maximum Likelihood (ML) phylogenetic tree of single‐copy orthologous proteins from the genera *Starmera*, *Phaffomyces*, *Cyberlindnera*, *Barnettozyma* and *Wickerhamomyces*, with *Komagataella pastoris* used as an outgroup. The genus *Wickerhamomyces* (pink), part of the family *Wickerhamomycetaceae*, is polyphyletic and interspersed among other genera from the family *Phaffomycetaceae*. Right: High variation is observed in the presence of transposable elements (TEs), secreted proteins, putative effectors, putative CAZymes and proteases. The number of TEs and codon usage patterns vary greatly between species and do not correlate with phylogenetic relationships.

We next compared the synteny of genes from our chromosome‐level assembly with high‐continuity genome assemblies of other *Barnettozyma* species to assess whether the high synteny observed within *B. californica* also extends to other species in the genus. Our results show that this is not the case. Instead, we find a low level of synteny between different *Barnettozyma* species. The closely related *B. populi* shows a relatively high level of synteny, with few inversions and translocations. In contrast, more distantly related species show much lower levels of synteny (Figure 4C and Figure S4B,C). We, therefore, conclude that synteny across species within the *Barnettozyma* genus is generally low. We also compared the genome functional composition of 74 additional species within the *Phaffomycetales* order, including *Komagataella pastoris* as an outgroup to discern patterns of evolution and adaptation in the family. Notably, *Komagataella* was previously classified within the *Phaffomycetaceae* (Kurtzman 2011), but genome‐scale analyses now place it under the *Pichiales*, making it a suitable outgroup for this study (Shen et al. 2018; Groenewald et al. 2023). To correlate phylogenetic relationships with genome functional composition, we reconstructed a maximum likelihood phylogenetic tree based on concatenated protein sequences of single‐copy orthologous genes (a total of 657 genes) (Figure 5, right). To ensure comparability between the species, we functionally reannotated each of the assemblies using the same bioinformatics pipeline employed for the annotation of *B. californica* MYf642. Our results confirm the monophyletic origins of the genera within the *Phaffomycetaceae*, specifically *Barnettozyma*, *Cyberlindnera*, *Phaffomyces* and *Starmera*. However, the *Wickerhamomycetaceae*, which contains the genus *Wickerhamomyces*, does not appear monophyletic and is interspersed among species of the *Phaffomycetaceae*. Although outside the main focus of this study, it is important to note that the non‐monophyletic characteristics of the *Wickerhamomyces* genus, which we here inferred from 657 orthologous genes, contrast with earlier analyses based on internal transcribed spacer (ITS) and large ribosomal subunit (LSU) sequences (Arrey et al. 2021; Nundaeng et al. 2021) or elongation factor‐1α (EF‐1α) and small ribosomal subunit (SSU) sequences (Kobayashi et al. 2017). The functional genome composition of *B. californica* MYf642 is similar to that of other species within the genus *Barnettozyma* and other species in the *Phaffomycetales*. Despite a high variation in certain genomic features, such as transposable element content (ranging from 0.2% to 7.4%), secreted proteins (239–482), effector candidates (58–248), proteases (175–238) and CAZymes (112–172), none of these variations appear to correlate with phylogenetic relationships, nor does *B. californica* MYf642 stand out as an extreme in any category. Additionally, we observed high variation in codon usage bias, as predicted by gene‐wise relative synonymous codon usage (RSCU), and the predicted numbers of tRNAs. However, neither codon usage bias nor tRNA numbers appeared to be correlated or follow any phylogenetic pattern, and again, nor does *B. californica* MYf642 stand out as an extreme. In conclusion, the functional genome composition of *B. californica* MYf642 is broadly similar to other related species, despite the observed variations in certain genomic traits.

### 2.7 *B. californica* MYf642 Contains Unique Genes Associated With Terpenoid Metabolism and Transport

**FIGURE 6:**
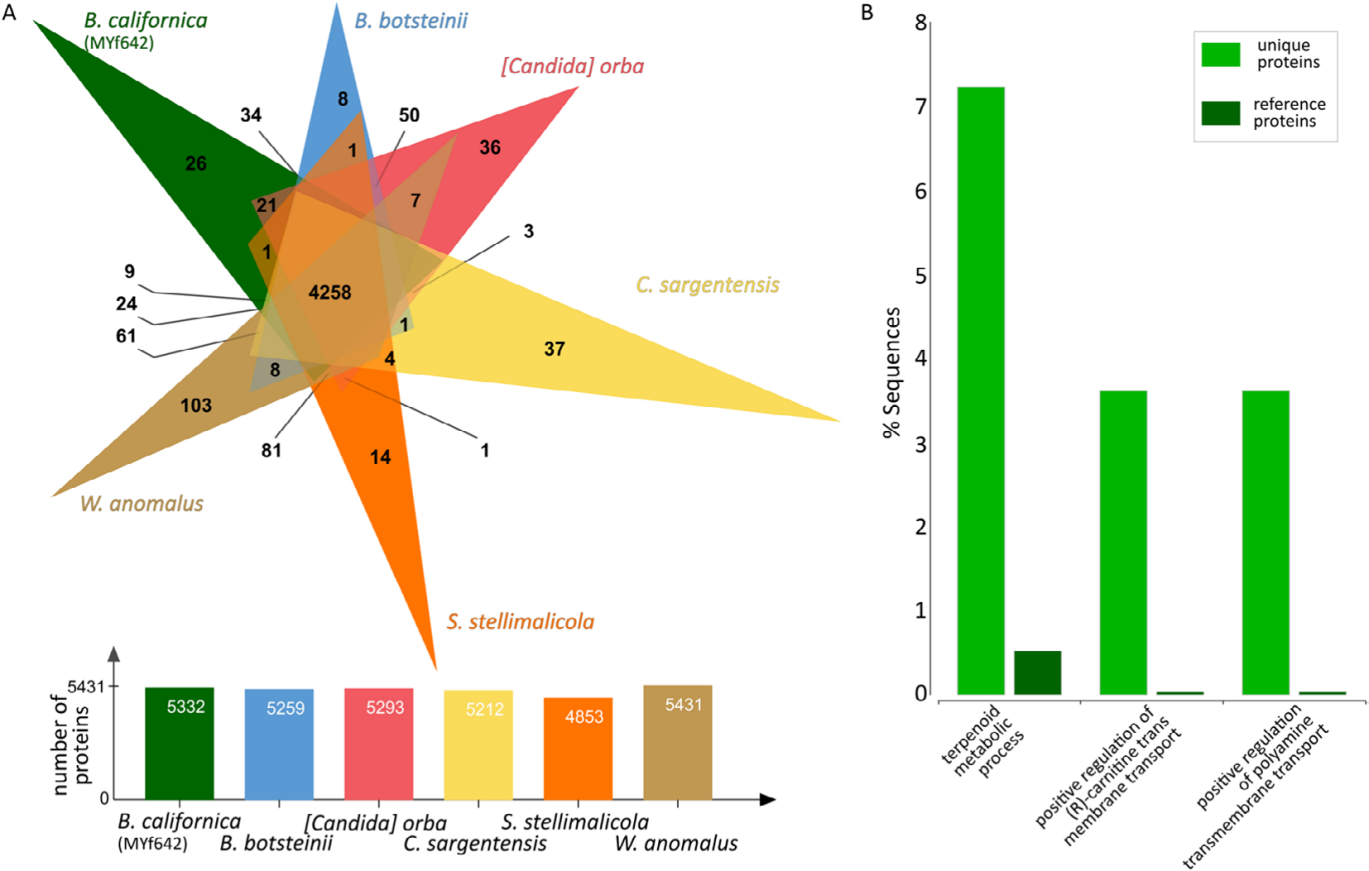
Identification of proteins restricted to *B. californica* MYf642 and associated GO terms. (A) Venn diagram of orthologous proteins among representative species of the Phaffomycetales compared to the *B. californica* MYf642 isolate, identifying 28 clusters with a total of 83 proteins that are restricted to *B. californica* MYf642 and do not have orthologs in the other representative species. (B) Results of the GO term enrichment analysis for the 83 proteins restricted to *B. californica* MYf642 compared to the reference set (all proteins encoded by the *B. californica* MYf642 genome), with significance thresholds of *p* = 0.05 and FDR = 0.1.

We next compared the gene composition of *B. californica* MYf642 with representative members of other genera in the *Phaffomycetales* for which high‐continuity genome assemblies are available. A total of 4258 orthogroups were shared across all six species, while 28 orthogroups were unique to *B. californica* MYf642 (Figure 6A,B). Based on these 28 orthogroups and functional gene annotations (Table S6), we conducted a GO‐term enrichment analysis. This analysis identified several significantly over‐represented GO terms, including ‘terpenoid metabolic processes’, ‘positive regulation of (R)‐carnitine transmembrane transport’ and ‘polyamine transmembrane transport’. Interestingly, terpenoids and terpenes, form the largest group of secondary metabolites in fungi (González‐Hernández et al. 2023). The increased *C. elegans* fitness for some *C. elegans* genotypes, observed when *B. californica* MYf642 and *E. coli* OP50 are both available as a food source, may be attributed to fungal compounds such as secondary metabolites. These specific compounds could be limited in pure bacterial cultures. Additionally, we tested whether genes involved in nitrification and denitrification, previously reported in the *B. californica* K1 strain—specifically amoA, nirK, nosZ (Fang et al. 2021)—were present in *B. californica* MYf642. However, none of these genes were detected by BLAST searches using the sequences described in (Fang et al. 2021). Therefore, we conclude that *B. californica* MYf642 does not possess the genetic capacity for heterotrophic nitrification‐aerobic denitrification.

## 3 Discussion

Here, we show that the fungus *B. californica* , which is found in diverse environments and was isolated as a putatively beneficial microbiome member of *C. elegans* from a mesocosm experiment, is ingested and used as food by *C. elegans* . On its own, it appears to be a poor food source—potentially due to its large cell size—leading to increased *C. elegans* foraging behaviour. However, when combined with a bacterial food source, it can have a positive fitness effect and reduce foraging activity. Furthermore, we report the chromosome‐level assembly of the *B. californica* MYf642 genome, which includes a novel type of telomeric repeat. The genome is highly compact and does not exhibit any distinct features that set it apart from other *Phaffomycetales* yeasts and that could thus explain its interaction with *C. elegans* and its bacterial food.

The interaction between *C. elegans* and fungi is poorly understood, and the extent to which fungi contribute to the *C. elegans* diet remains unknown. Our study is one of the few that demonstrates that fungi can serve as a food source for *C. elegans* . Similarly, the basidiomycete yeasts *Cryptococcus kuetzingii* and *C. laurentii* have been used as *C. elegans* food sources in the lab (Mylonakis et al. 2002). However, for neither *Cryptococcus* species was a direct association with *C. elegans* shown, nor were they isolated from a *C. elegans* environment. Notably, in our experiments, a positive effect on *C. elegans* population growth was observed only when *B. californica* MYf642 was present alongside *E. coli* OP50, and this effect was dependent on the *C. elegans* genotype. In some genotypes, the combination of fungus and bacteria as food increased nematode fitness, while in others, it reduced it—indicating a genotype‐dependent response to the fungus‐bacterium diet. One possible explanation is the relatively large cell size of *B. californica* , which may hinder its ingestion by early larval stages—possibly dependent on the *C. elegans* genotype. In such cases, an additional food source like *E. coli* may be necessary to support proper worm development. In addition to directly affecting *C. elegans* biology, *B. californica* MYf642 may influence it indirectly via interactions with *E. coli* OP50. In general, fungi and bacteria that share habitats can interact through the production of antibiotics, signalling molecules and other compounds, such as siderophores that manipulate nutrient availability (Deveau et al. 2018; Scherlach and Hertweck 2020; Pierce et al. 2021). Currently, the mechanism underlying the positive effects of *B. californica* MYf642 in the presence of *E. coli* OP50 remains unclear. One possibility is that the fungus produces compounds that are limited in a pure bacterial culture. Many fungi are known to produce secondary metabolites with great structural diversity (Macheleidt et al. 2016), which may be beneficial to *C. elegans* . Alternatively, and not mutually exclusively, *B. californica* may induce changes in *E. coli* that are beneficial to *C. elegans* . In any case, our results suggest that this effect is dependent on the genotype of the nematode. Notably, in this tripartite interaction ( *C. elegans* — *B. californica* MYb642— *E. coli* OP50), we varied only the *C. elegans* genotype in our experiments. It would be highly interesting to explore whether this interaction is also influenced by variation in the bacterial and fungal components—for example, by using the CeMbio bacterial microbiome or by including additional fungi naturally associated with *C. elegans* . We also found that the foraging behaviour of *C. elegans* was affected by *B. californica* MYf642 when present in combination with *E. coli* OP50, and this effect was again dependent on the *C. elegans* genotype. It is known that genetically different *C. elegans* strains vary in their lawn‐leaving behaviour in response to food bacteria and pathogens (Reddy et al. 2009; Bendesky et al. 2011; Nakad et al. 2016). Leaving a microbial lawn can be an avoidance behaviour towards certain pathogens, such as *Serratia marcescens* (Pradel et al. 2007) or *B. thuringiensis* (Schulenburg and Müller 2004; Hasshoff et al. 2007; Nakad et al. 2016). However, *C. elegans* can also distinguish between high‐ and low‐quality food bacteria. Worms tend to search for high‐quality food that supports growth, while they avoid or abandon low‐quality food (Shtonda and Avery 2006). Thus, the increased foraging behaviour in the presence of *B. californica* MYf642 indicates that it is a poor food source, while the reduced foraging behaviour of some *C. elegans* strains when present with the mixture of fungi and bacteria indicates that this is a good food source. In microbial lawn‐leaving assays, nematodes often return to the food multiple times within minutes to hours, which might explain the differences between our 2 and 24‐h data in the lawn‐leaving assay. At present, we can only speculate about why the *C. elegans* strains JU258 and EG4725 exhibited a more beneficial interaction with the microbial mixture than the other strains tested in our experiment. Although some studies have investigated aspects of their microbiome (Zhang, Weckhorst, et al. 2021) and starvation resistance (Webster et al. 2022), the available data do not reveal a consistent or distinctive profile for these two isolates compared to other natural isolates. JU258 originates from the island of Madeira, while EG4725 is from mainland Portugal (Crombie et al. 2024). However, in the absence of a clear shared trait or genetic basis, the underlying cause of their enhanced interaction with the microbial mixture remains unresolved. A comprehensive genome‐wide association study (GWAS), incorporating additional mapping populations from the CaeNDR (Crombie et al. 2024) followed by functional validation, will likely be necessary to identify and confirm the genetic factors involved.

The genomes of fungi are shaped by interactions with other organisms that share the same habitat, including other microorganisms. The discovery of penicillin by Alexander Fleming in 1929 was the first indication that fungi manipulate their microbial environment through what we now recognise as secondary metabolites. Fungi also produce small secreted proteins, so‐called effectors. Until recently, the role of effectors in plant‐associated fungi was largely thought to be limited to interactions with the host and the suppression of the host immune system (Jones and Dangl 2006). More recently, effectors have been shown to influence the bacterial and fungal microbiome of plants, suggesting that this may have been their ancestral function (Snelders et al. 2018, 2020, 2021). Although we were unable to identify any unique secondary metabolite clusters or effectors in the genome of *B. californica* MYf642 that might explain the fungus's positive impact—both in this study and in the mesocosm study (Petersen et al. 2023)—this does not rule out the existence of yet‐undiscovered genetic components involved in these interactions. Additionally, it is puzzling that we did not find substantial genetic overlap with the termite‐associated *B. botsteinii* (Arrey et al. 2021). Thus, the fungal genetic components underlying the interaction with *C. elegans* and *E. coli* OP50 remain unknown.

In conclusion, the microbial lawn‐leaving assay and the population growth assay demonstrated that *B. californica* MYf642 affects *C. elegans* behaviour and fitness when presented jointly with bacteria, in a genotype‐dependent manner. The use of genetically diverse mapping strains from the CaeNDR has allowed us to identify these patterns, which are not apparent in the standard laboratory N2 strain. The interaction between *C. elegans* , *B. californica* MYf642 and *E. coli* OP50 that we present here is an intriguing example of the complexity and multi‐level nature of interactions between fungi, bacteria and animal hosts – for which *C. elegans* is one of the ideal model organisms. Alongside the established CeMbio community, this study provides a valuable foundation for further exploring how interactions between fungi and bacteria within the *C. elegans* microbiome might shape community structures and influence host biology, while also exemplifying the importance of isolating fungal components from the *C. elegans* microbiome. Future research will focus on uncovering the genetic basis of this interaction in both *C. elegans* and *B. californica* MYf642 as well as exploring the interactions between the natural bacterial and fungal microbiomes of *C. elegans* .

## 4 Methods and Materials

### 4.1 Microbial and *C. elegans* Strains

*E. coli* OP50, *E. coli* OP50::dTomato and *B. californica* MYf642 were cultured overnight in a shaking incubator (180 rpm) in lysogeny broth (LB) medium at 28°C. We used the 12 *C. elegans* strains CB4856, CX11314, DL238, LKC34, ED3017, EG4725, JT11398, JU258, JU775, MY16, MY23 and N2 from a mapping population provided by the *Caenorhabditis* Natural Diversity Resource (CaeNDR) (Cook et al. 2017; Crombie et al. 2024). Additionally, the *C. elegans strain* dkIs37[*act‐5p::GFP::pgp‐1*] was used to locate stained cells of *B. californica* MYf642 in the *C. elegans* intestine. All *C. elegans* strains were maintained on nematode growth medium (NGM) on *E. coli* OP50.

### 4.2 Isolation of *Barnettozyma californica* MYf642

We isolated *B. californica* MYf642 from a 100‐day‐old laboratory compost mesocosm, originating from a long‐term evolution experiment with *C. elegans* and its associated microbes. Worms from this 100‐day‐old laboratory compost showed improved fitness and enrichment of *Barnettozyma* in a follow‐up common garden experiment when exposed to the microbial community from day 100 (Petersen et al. 2023). To isolate *Barnettozyma californica* MYf642, a random compost sample was mixed with 25 mL M9 with 0.025% Triton X‐100 (M9‐T). A 300 μL aliquot of this mixture was homogenised with 10 zirconia beads (1 mm) in a Bead Ruptor 96 (OMNI International, Kennesaw, GA, USA) at 30 Hz for 3 min, then frozen in 10% dimethyl sulfoxide (DMSO) at −80°C. Thawed material was serially diluted (10^−1^ to 10^−4^) in sterile phosphate buffered saline (PBS), and 50 μL of each dilution was plated onto potato dextrose agar (39 g/L, Carl Roth, Karlsruhe, Germany). Plates were incubated at room temperature (RT) for 10 days. Morphologically distinct colonies were picked upon appearance and purified by re‐culturing at least three times. Pure isolates were grown in LB medium on a circular shaker for 2 days at 25°C and preserved in 35% glycerol at −80°C. Crude DNA was extracted by adding 5 μL liquid culture to 19.5 μL 5× PCR buffer with 0.5 μL proteinase K (20 mg/mL), followed by freezing at −80°C for 30 min, digestion at 50°C for 1 h and heat inactivation at 95°C for 15 min. For identifying yeasts and fungi, 1 μL crude DNA served as a template in a 50 μL PCR reaction using DreamTaq DNA polymerase (Thermo Fisher Scientific, Darmstadt, Germany) to amplify the internal transcribed spacer (ITS) region with the primers ITS1f (5′‐CTTGGTCATTTAGAGGAAGTAA‐3′) and ITS4r (5′‐TCCTCCGCTTATTGATATGC‐3′) (White et al. 1990; Gardes and Bruns 1993).

An overnight culture of *B. californica* MYf642 in LB medium, grown at 28°C and 200 rpm, was used for DNA isolation following a modified version of the cetyltrimethylammonium bromide (CTAB) DNA extraction protocol (Plissonneau et al. 2016). Briefly, the cells were harvested by centrifugation, washed twice and 400 μL of the cell pellet was crushed using a mortar and pestle with liquid nitrogen. Following extraction with CTAB, DNA was isolated twice using phenol:chloroform:isoamylalcohol and precipitated with isopropanol. The DNA was washed three times with ethanol before being resuspended in 1× TE buffer. Sequencing was performed using the PacBio Revio platform at BGI Techsolutions (Hong Kong), resulting in 308,037 CCS reads with a mean length of 17,689 bp.

### 4.3 Cell Size Measurement

Cultures of *B. californica* MYf642 and *E. coli* OP50 were grown in LB medium for 24 h at 28°C under continuous shaking (180 rpm). Representative images were acquired by microscopy (Zeiss Axio Observer Z.1, Carl Zeiss AG, Jena, Germany) at 100× magnification. Morphometric analysis of 60 individual cells per strain was performed in ImageJ (version 2.14.0/1.54i), quantifying cell length and width.

### 4.4 Calcofluor White Staining

Calcofluor white stain is a fluorochrome that binds to cellulose and chitin in cell walls of fungi. The fourth larval stage (L4) of the *C. elegans* strain dkIs37[*act‐5p::GFP::pgp‐1*] expressing PGP‐1::GFP in the apical intestinal membrane (Sato et al. 2007) was transferred to NGM plates inoculated with *B. californica* MYf642. After 24 h, adult worms were washed from the plates with M9‐buffer containing 0.3% Tween20 (M9‐T20). 500 μL of the worm‐containing M9‐T20 was added to a 2‐mL tube, and 500 μL of 10 mM tetramisole was added to stop the ingestion and excretion of microbes. After about 2 min, the pellet of immobilised worms was transferred to a new tube. The worms were fixed and stained by adding 250 μL of calcofluor white staining solution consisting of equal parts calcofluor white stain and ethanol. After 3 h, the staining solution was removed; the worms were washed three to four times with M9‐T20 and then examined for stained *B. californica* MYf642 in their intestine using confocal laser scanning microscopy (ZEISS LSM 880).

### 4.5 Microbial Colonisation Assay

Microbial colonisation rates were assessed as previously described (Zimmermann et al. 2020; Pees et al. 2021). Briefly, L4 larvae of the *C. elegans* strain N2 were transferred to 6‐cm NGM plates inoculated with either OP50::dTomato, *B. californica* MYf642, or an equal mixture of both (OD_600_ = 10). After 24 h, adult worms were collected using M9‐T, washed five times and 100 μL worm pellet was transferred to a new sterile microtube. Here, 100 μL 10 mM tetramisole hydrochloride was added. Worms were surface‐sterilised using M9‐T with 2% sodium hypochlorite, then washed twice with PBS including 0.025% Triton X‐100 (PBS‐T). Surface‐sterilised worms were transferred to a new sterile microtube, counted and the volume adjusted to 400 μL with PBS‐T. After addition of 1 mm zirconia beads, 100 μL supernatant was collected and 50 μL plated on LB agar to verify sterilisation. Worms in the remaining 300 μL were homogenised using a Bead Ruptor 96 (OMNI International, Kennesaw, GA, USA) for 3 min at 30 Hz. Undiluted and diluted (10^−1^ and 10^−2^) homogenates were plated (50 μL each) on LB agar. After 2–3 days at RT, colonies were counted. For worms exposed to mixed microbial diets, colonies were distinguished based on morphology or fluorescence and counted on the same plate. Colony‐forming units (CFU) per worm were calculated for each microbe. The experiment was performed using a letter code and in randomised order to avoid observer bias.

### 4.6 Population Growth Assay

The population growth rate is a measure for worm fitness as it combines information on the developmental time, reproductive rate and survival similar to the way described in (Petersen et al. 2015; Dirksen et al. 2016). To start the assay, three *C. elegans* L4 larvae were picked to a 1‐mL lawn of either *B. californica* MYf642, *E. coli* OP50 or an equal mixture of both at OD_600_ 10 on 9 cm peptone‐free medium (PFM) plates. After 5 days at 20°C, the worms were washed from the plates to 15 mL Falcon tubes using 5 mL M9‐T and frozen at −20°C until the worms were counted. All worms (including all larval stages and adults, but not eggs) contained in subsamples of 5 to 100 μL were counted; the worm count was then extrapolated to the total amount of worm‐containing buffer to obtain the total number of worms. Three subsamples (technical replicates) were counted per biological replicate, from which a mean value was calculated to account for variations in worm numbers. The offspring number was divided by the three original worms to obtain the offspring per worm produced in 5 days. The experiment was performed using a letter code and in randomised order to avoid observer bias.

### 4.7 Development Assay

To assess whether *C. elegans* can complete its full life cycle (i.e., develop from the first larval stage (L1) to adults) on *B. californica* MYf642, we placed approximately 150 bleached and synchronised L1 larvae of the N2 wildtype strain on 6‐cm PFM plates seeded with either *E. coli* OP50, *B. californica* MYf642 or an equal mixture of both (OD_600_ = 10). After 72 h at 20°C, worms were washed off the plates and the proportion of adults was determined by counting both adult and larval stages. Remaining plates were kept at 20°C and monitored daily by visual inspection. The experiment was performed using a letter code and in randomised order to avoid observer bias.

### 4.8 Microbial Lawn Leaving Assay

*C. elegans* can perceive the quality of its food as well as the presence of pathogens and adapt its behaviour accordingly (Shtonda and Avery 2006; Meisel and Kim 2014). To determine the behaviour of *C. elegans* towards *B. californica* MYf642, we performed a microbial lawn leaving assay. Approximately 30 synchronised L4 worms were pipetted to a 60 μL microbial spot of *B. californica* MYf642, *E. coli* OP50 or an equal mixture of both adjusted to OD_600_ 10 on 6‐cm PFM plates. The number of worms on and outside the spot was counted after 2 and 24 h to analyse the early and late lawn leaving behaviour. The escape rate was then calculated by dividing the number of worms outside the microbial spot by the total number of counted worms. The experiment was performed using a letter code and in randomised order to avoid observer bias.

### 4.9 Choice Assay

We tested the influence of *B. californica* MYf642 on choice behaviour, following the approach used in previous experiments (Shtonda and Avery 2006; Petersen et al. 2021). Approximately 25 synchronised L4 worms (strains N2 or JU258) were pipetted centrally between 30 μL spots of a mixture of *E. coli* OP50 and *B. californica* and *E. coli* OP50 only on 6‐cm NGM plates. To examine the early and late choice behaviour, the number of worms residing on each microbe was determined after 2, 6 and 24 h. We calculated a choice index = (Number of worms on OP50‐*Barnettozyma* mix−Number of worms on OP50)/(Total number of worms on both microbes). A choice index of 1 indicates the choice of the mixed microbial lawn over OP50, a choice index of −1 indicates the choice of OP50 over the mixed lawn, and a choice index of 0 indicates equal choice of both lawns. The experiment was performed using a letter code and with randomised order of the microbial spots on the plates to avoid observer bias.

### 4.10 Bioinformatics Analysis

#### 4.10.1 Genome Assembly

The code used for all subsequent bioinformatics analyses has been deposited here: https://github.com/michaelH‐git/BarnettozymaCalifornica_genomeAnalysis.git. Briefly, assemblies were generated using Canu (2.2) (Koren et al. 2017), HiFiasm (0.19.9‐r616) (Cheng et al. 2021) and Flye (2.9.3‐b1797) (Kolmogorov et al. 2019). The assembly quality was checked using QUAST (v5.2.0) (Mikheenko et al. 2018). After visual inspection, the Canu‐based assembly was selected for all subsequent analyses. The quality of this assembly was further evaluated using Tapestry (1.0.1) (Davey et al. 2020), and contigs that showed no coverage by the original reads were discarded (40 contigs, totalling 1.3 Mb). Of the remaining nine contigs, the smallest one (TIG20) showed synteny to mitochondrial genomes. This sequence was further polished and annotated using MitoHiFi (3.2.1) (Uliano‐Silva et al. 2023), resulting in a 48,643 bp circular contig representing the mitogenome. For the remaining seven contigs representing the nuclear genome, telomere sequences were identified using TelFinder (Sun, Wang, et al. 2023) and the quality of the genome was rechecked using Tapestry.

#### 4.10.2 Functional Annotation of Genomes

The list and accessions of the genome assemblies included in this study are given in Table S7. Genomes were functionally annotated using the following tools. Transposable and repetitive elements were identified using EDTA (2.2.0) (Ou et al. 2019). In cases where no transposable elements (TEs) were detected, repetitive sequences were called using EarlGrey (4.4.4) (Baril et al. 2024). These annotations were used to softmask the genomes for gene annotation. Genes were annotated using BRAKER3 (3.0.8) (Gabriel et al. 2024) with protein evidence from OrthoDB11 restricted to fungi. Secondary metabolite clusters were annotated using antiSMASH (7.1.0) (Blin et al. 2021), and functional annotation of predicted proteins was performed using eggNOG‐mapper (2.1.12) (Cantalapiedra et al. 2021) and InterProScan (Blum et al. 2021). Genome completeness was assessed with BUSCO (5.7.1) (Simão et al. 2015), while tRNAs were predicted using tRNAscan‐SE (2.0.12) (Chan et al. 2021). Codon usage was estimated by determining the gene‐wise relative synonymous codon usage (gRSCU) with BioKit (Steenwyk and Buida 2022). Putatively secreted proteins were identified using SignalP (5.0b) (Almagro Armenteros et al. 2019), and putative effectors were predicted using EffectorP3 (3.0) (Sperschneider and Dodds 2022). Putative CAZymes (carbohydrate‐active enzymes) were identified using dbCAN (Zhang et al. 2018). All these annotations were combined using Funannotate (Funannotate 2017). For *B. californica* MYf642, the functional annotation was further improved using Blast2GO (Conesa and Götz 2008). Fisher's Exact test for GO‐term enrichment was conducted using this improved annotation in Blast2GO.

#### 4.10.3 Phylogenetic Analysis

Identification of single copy orthologs for all genomes of the phylogenetic analysis was conducted using Orthofinder (Emms and Kelly 2019) restricted to one transcript for each gene. All single copy orthologous proteins shared among all species (657) were concatenated and aligned using MAFFT and the phylogenetic tree determined by IQTree using the LG + I + G model and generating a consensus tree of 1000 bootstrapings (Minh et al. 2020). Determination of orthologous proteins between *B. californica* MYf642, *B. botsteinii* (GCA_020280145.1), *C. sargentensis* (GCA_020995425.1), *S. stellimalicola* (GCA_030580055.1), *W. anomalus* (GCA_019321675.1) and *[Candida] orba* (GCA_003708145.3) to represent all genera within the phylogenetic analysis was performed using OrthoVenn3 (Sun, Lu, et al. 2023). GO term enrichment analysis was performed on those genes unique to *B. californica* MYf642. Synteny analysis was conducted using GENESPACE (v1.3.1) (Lovell et al. 2022).

## Author Contributions

**Carola Petersen:** investigation, formal analysis, writing – original draft, writing – review and editing. **Hanne Griem‐Krey:** investigation, formal analysis, writing – original draft, writing – review and editing. **Christina Martínez Christophersen:** investigation. **Hinrich Schulenburg:** conceptualization, supervision, writing – review and editing. **Michael Habig:** conceptualization, investigation, formal analysis, writing – original draft, writing – review and editing.

## Conflicts of Interest

The authors declare no conflicts of interest.

## Supporting information


**Figure S1:** *B. californica* MYf642 in combination with *E. coli OP50* as food results in development comparable to that on *E. coli* OP50 alone and in a strain‐dependent effect on the behavioural response after 2 h. (A) Proportion of adult *C. elegans* N2 after 72 h fed with either *E. coli* OP50 (grey), *E. coli* OP50 combined with *B. californica* MYf642 (dark green) or *B. californica* MYf642 alone (light green). No adults were observed with *B. californica* MYf642 as the sole food source, whereas the mixed diet supported development comparable to the *E. coli* OP50 control. *n* = 8. (B) The figure shows the behavioural response of the indicated *C. elegans* strains on *E. coli* OP50 (grey), *E. coli* OP50 combined with *B. californica* MYf642 (dark green) and *B. californica* MYf642 alone (light green). *B. californica* MYf642 as the sole food source results in a higher number of worms outside the microbial lawn after 2 h for all strains (compared to *E. coli* OP50 as sole food source), with the exception of CX11314. When combined with *E. coli* OP50, *B. californica* MYf642 leads to genotype dependent variation in the fraction of worms outside the microbial lawn. Significant differences (determined by the Wilcoxon rank sum test with Holm correction for multiple testing) compared to *E. coli* OP50 as the sole food source are indicated as follows: *p* < 0.05 (*), *p* < 0.01 (**), *p* < 0.001 (***), ns = non‐significant. *n* = 8 biological replicates.

**Figure S2:** Correlation between lawn‐leaving behaviour and population growth. The relative number of offspring per worm for each *C. elegans* strain on the mixed microbial lawn (normalised to the *E. coli* OP50 control) is plotted against the relative fraction of worms outside the microbial lawn for each *C. elegans* strain (normalised to the *E. coli* OP50 control). A linear regression line is indicated by the dashed line. *n* = 8 biological replicates.

**Figure S3:** Choice behaviour of *C. elegans* N2 and JU258 towards *E. coli* OP50 alone and *E. coli* OP50 in combination with *B. californica* MYf642. Approximately 25 synchronised L4 larvae were transferred centrally between microbial spots of *E. coli* OP50 and *E. coli* OP50 in combination with *B. californica* MYf642. The choice behaviour was evaluated after 2, 6 and 24 h. A choice index of +1 indicates choice of the combined microbes, a choice index of −1 indicates choice of *E. coli* OP50, and a choice index of 0 (indicated by dashed line) indicates equal choice of both sides. Each dot represents one replicate, Wilcoxon signed rank test (FDR‐corrected for multiple comparisons), *n* = 22.

**Figure S4:** Network phylogeny of species within the genus *Barnettozyma* and details on synteny within *B. californica* support chromosome‐level assemblies and extensive structural variation within the *Barnettozyma* genus. (A) NeigborNet phylogeny of 2710 single copy orthologous proteins of species within the genus *Barnettozyma* and with *E. anomalus* as an outgroup. The isolate *B. californica* MYf642 falls within the species *B. californica* . (B) Details of orthologous proteins based synteny for TIG09 and TIG15 (left) and TIG19 (right) show that the apparent structural variation is unique to the *B. californica* strain PB4207, where it is located at the end of a contig. All other previously published assemblies show no structural variation compared to *B. californica* MYf642. Hence, this apparent structural variation in *B. californica* strain PB4207 could be due to a misassembly. (C) Details of synteny for TIG01 (left) and TIG10 (right) demonstrate extensive structural variation, which increases with phylogenetic distance. Panels (B) and (C) show details from Figure 4 panel (B) and (C), respectively.

**Table S1:** Phenotypic raw data.

**Table S2:** Statistical analysis of phenotypic data.

**Table S3:** Overview of *B. californica* MYf642 genome assembly and characteristics.

**Table S4:** Comparison of the functional annotations of the genomes used for the phylogenetic analysis.

**Table S5:** Overview of the transposable elements (TE) and repeat elements (RE) in the *B. californica* MYf642 assembly.

**Table S6:** Functional annotation of proteins encoded by *B. californica* MYf642.

**Table S7:** Genome assembly accessions included in this study.

**Movie S1:** Feeding behaviour of *C. elegans* including ingestion, passage through the worm intestine and excretion of *B. californica* MYf642.

**Movie S2:** Feeding behaviour of *C. elegans* including ingestion, passage through the worm intestine and excretion of *B. californica* MYf642.

## Data Availability

Sequencing reads, genome assemblies and annotations for *B. californica* MYf642 are deposited using Bioproject PRJNA1224554.
