# Supplementary figures and images for: The Interaction Between the Nematode Caenorhabditis elegans and Its Coexisting Fungal Microbiome Member Barnettozyma californica
Source: Environ Microbiol Rep. 2025 Aug 19;17(4):e70177. doi: 10.1111/1758-2229.70177 (PMC12361814; doi:10.1111/1758-2229.70177)

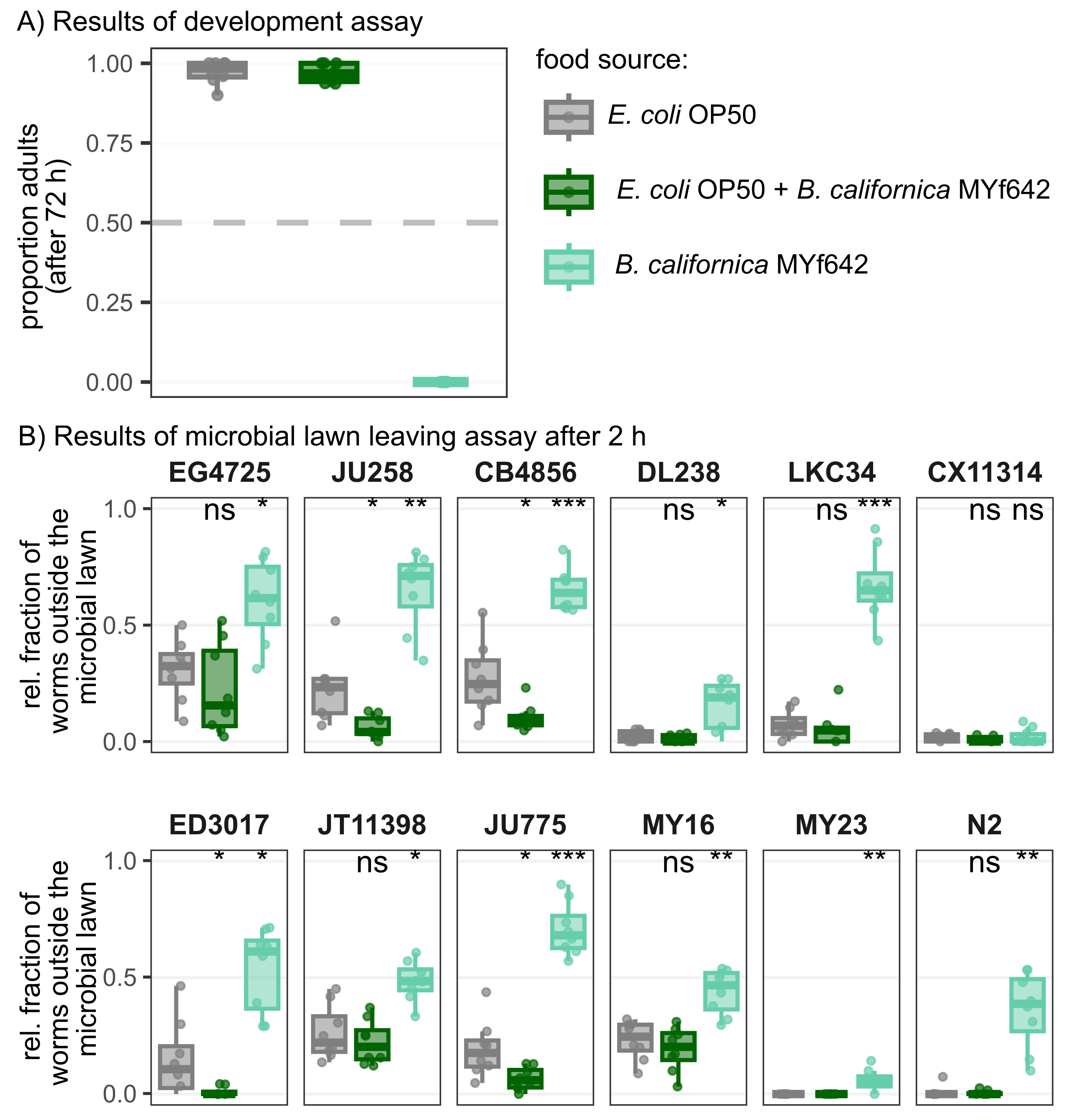

Supplement: Supplementary file 1 — Figure S1: B. californica MYf642 in combination with E. coli OP50 as food results in development comparable to that on E. coli OP50 alone and in a strain‐dependent effect on the behavioural response after 2 h. (A) Proportion of adult C. elegans N2 after 72 h fed with either E. coli OP50 (grey), E. coli OP50 combined with B. californica MYf642 (dark green) or B. californica MYf642 alone (light green). No adults were observed with B. californica MYf642 as the sole food source, whereas the mixed diet supported development comparable to the E. coli OP50 control. n = 8. (B) The figure shows the behavioural response of the indicated C. elegans strains on E. coli OP50 (grey), E. coli OP50 combined with B. californica MYf642 (dark green) and B. californica MYf642 alone (light green). B. californica MYf642 as the sole food source results in a higher number of worms outside the microbial lawn after 2 h for all strains (compared to E. coli OP50 as sole food source), with the exception of CX11314. When combined with E. coli OP50, B. californica MYf642 leads to genotype dependent variation in the fraction of worms outside the microbial lawn. Significant differences (determined by the Wilcoxon rank sum test with Holm correction for multiple testing) compared to E. coli OP50 as the sole food source are indicated as follows: p < 0.05 (*), p < 0.01 (**), p < 0.001 (***), ns = non‐significant. n = 8 biological replicates. [file EMI4-17-e70177-s012.png]

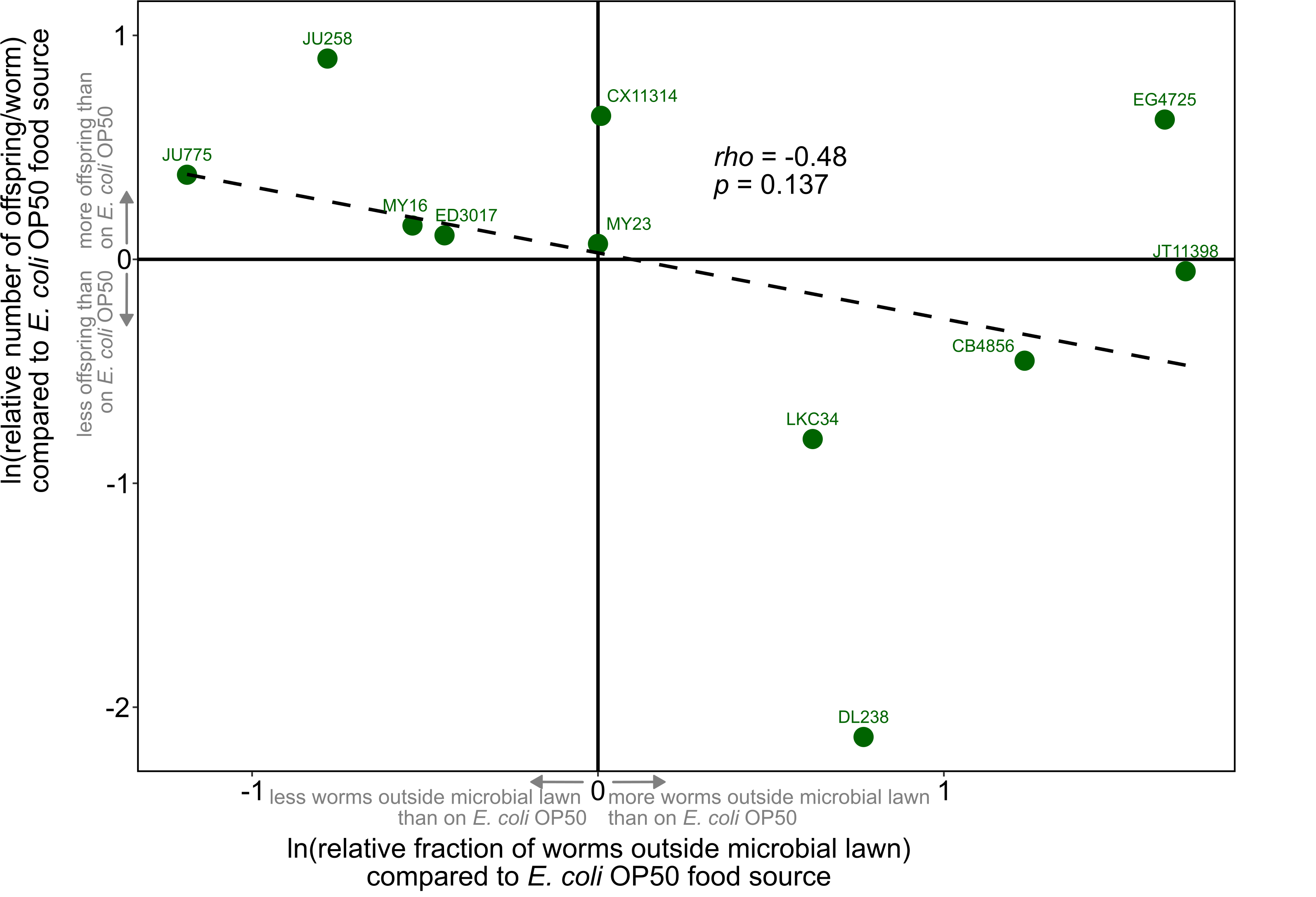

Supplement: Supplementary file 2 — Figure S2: Correlation between lawn‐leaving behaviour and population growth. The relative number of offspring per worm for each C. elegans strain on the mixed microbial lawn (normalised to the E. coli OP50 control) is plotted against the relative fraction of worms outside the microbial lawn for each C. elegans strain (normalised to the E. coli OP50 control). A linear regression line is indicated by the dashed line. n = 8 biological replicates. [file EMI4-17-e70177-s005.png]

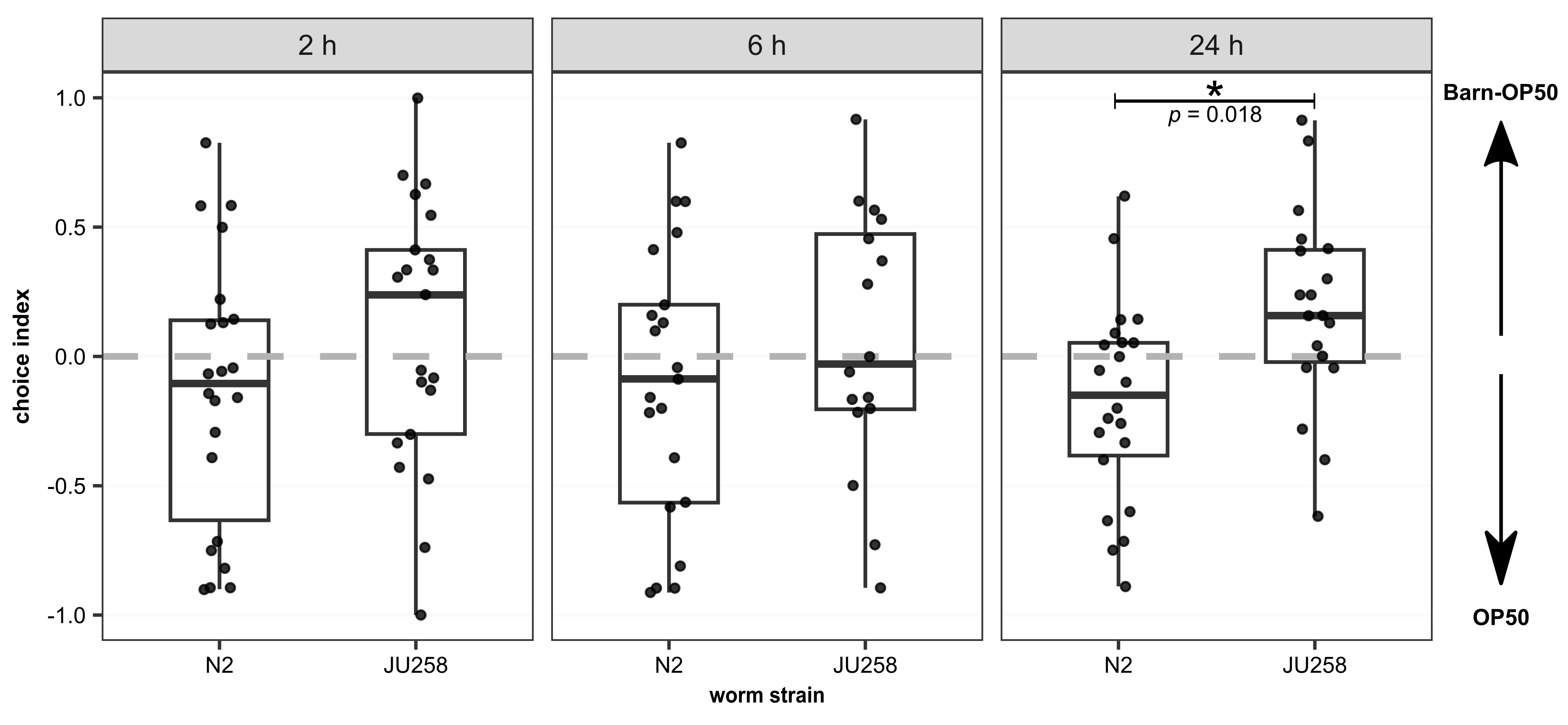

Supplement: Supplementary file 3 — Figure S3: Choice behaviour of C. elegans N2 and JU258 towards E. coli OP50 alone and E. coli OP50 in combination with B. californica MYf642. Approximately 25 synchronised L4 larvae were transferred centrally between microbial spots of E. coli OP50 and E. coli OP50 in combination with B. californica MYf642. The choice behaviour was evaluated after 2, 6 and 24 h. A choice index of +1 indicates choice of the combined microbes, a choice index of −1 indicates choice of E. coli OP50, and a choice index of 0 (indicated by dashed line) indicates equal choice of both sides. Each dot represents one replicate, Wilcoxon signed rank test (FDR‐corrected for multiple comparisons), n = 22. [file EMI4-17-e70177-s011.png]

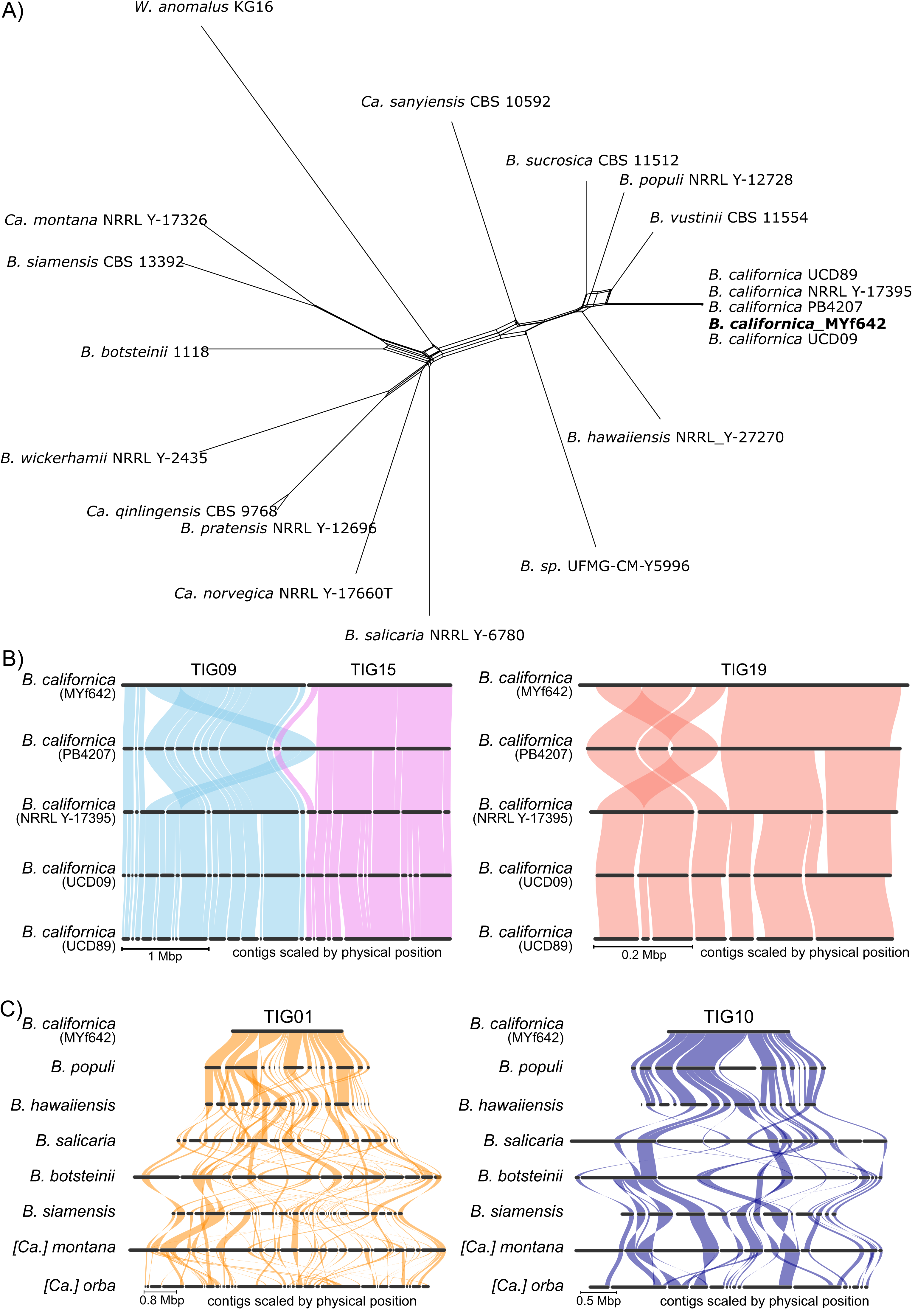

Supplement: Supplementary file 4 — Figure S4: Network phylogeny of species within the genus Barnettozyma and details on synteny within B. californica support chromosome‐level assemblies and extensive structural variation within the Barnettozyma genus. (A) NeigborNet phylogeny of 2710 single copy orthologous proteins of species within the genus Barnettozyma and with E. anomalus as an outgroup. The isolate B. californica MYf642 falls within the species B. californica . (B) Details of orthologous proteins based synteny for TIG09 and TIG15 (left) and TIG19 (right) show that the apparent structural variation is unique to the B. californica strain PB4207, where it is located at the end of a contig. All other previously published assemblies show no structural variation compared to B. californica MYf642. Hence, this apparent structural variation in B. californica strain PB4207 could be due to a misassembly. (C) Details of synteny for TIG01 (left) and TIG10 (right) demonstrate extensive structural variation, which increases with phylogenetic distance. Panels (B) and (C) show details from Figure 4 panel (B) and (C), respectively. [file EMI4-17-e70177-s007.png]
